# Serum Levels of Adipolin and Adiponectin and Their Correlation with Perinatal Outcomes in Gestational Diabetes Mellitus

**DOI:** 10.3390/jcm13144082

**Published:** 2024-07-12

**Authors:** Mihai Muntean, Vladut Săsăran, Sonia-Teodora Luca, Laura Mihaela Suciu, Victoria Nyulas, Claudiu Mărginean

**Affiliations:** 1 Departament of Obstetrics and Gynecology 2, University of Medicine Pharmacy Science and Technology George Emil Palade of Târgu Mureș, 540142 Târgu Mureș, Romania; munteanmihai@yahoo.com (M.M.); sonialuca11@gmail.com (S.-T.L.); marginean.claudiu@gmail.com (C.M.); 2 Departament of Neonatology, University of Medicine Pharmacy Science and Technology George Emil Palade of Târgu Mureș, 540142 Târgu Mureș, Romania; lauramihaelasuciu@yahoo.com; 3 Departament of Informatics and Medical Biostatistics, University of Medicine Pharmacy Science and Technology George Emil Palade of Târgu Mureș, 540142 Târgu Mureș, Romania; victoria.rus@umfst.ro

**Keywords:** adipolin, adiponectin, gestational diabetes mellitus, newborn, pregnancy

## Abstract

**Objectives**: This study aimed to investigate the serum level of adipolin and adiponectin in healthy pregnant women and pregnant women with gestational diabetes mellitus (GDM) during the second trimester, the prepartum period, and in the newborns of these patients. **Methods**: A total of 55 women diagnosed with GDM and 110 healthy pregnant women were included in this study. Pearson’s and Spearman’s correlation coefficients were calculated to determine the association of adipolin and adiponectin with anthropometric markers of obesity (body mass index (BMI), mid-upper arm circumference (MUAC), tricipital skinfold thickness (TST)), inflammation markers (neutrophil-to-lymphocyte ratio (NLR), platelet-to-lymphocyte ratio (PLR), C-reactive protein (CRP)), and maternal glucose homeostasis parameters (fasting glucose, insulin, C peptide, glycosylated hemoglobin A1c (HbA1c), Insulin Resistance—Homeostatic Model Assessment (IR HOMA)). **Results**: There were no statistical differences between the adipolin value in patients with GDM compared to healthy patients (*p* = 0.65 at diagnosis and *p* = 0.50 prepartum) and in newborns from mothers with GDM compared to healthy mothers (*p* = 0.24). Adipolin levels are significantly higher in patients with GDM who gave birth via cesarean section (*p* = 0.01). In patients with GDM, the adipolin level correlates positively with HgA1c in the prepartum period. We found a positive correlation between the maternal adipolin values at diagnosis and prepartum and neonatal adipolin (respectively: r = 0.556, *p* = 0.001; r = 0.332, *p* = 0.013). Adiponectin levels were significantly lower in patients with GDM at diagnosis and prepartum (*p* = 0.0009 and *p* = 0.02), but their levels increased prepartum (5267 ± 2114 ng/mL vs. 6312 ± 3150 ng/mL *p* = 0.0006). Newborns of mothers with GDM had lower adiponectin levels than newborns of healthy mothers (*p* < 0.0001). The maternal adiponectin value correlates negatively with maternal BMI, MUAC, and IR HOMA in both groups at diagnosis and prepartum. There were no differences between the groups in terms of cesarean rate (*p* > 0.99). The relative risk of occurrence of adverse events in patients with GDM compared to healthy ones was 2.15 (95% CI 1.416 to 3.182), and the odds ratio for macrosomia was 4.66 (95% CI 1.591 to 12.69). **Conclusions**: There was no difference in adipolin levels between mothers with GDM and healthy mothers during the second trimester and the prepartum period. Adipolin is known to enhance insulin sensitivity and reduce inflammation, but unlike adiponectin, it does not appear to contribute to the development of GDM.

## 1. Introduction

GDM is defined as any degree of glucose intolerance with onset or first recognition during pregnancy [1], diagnosed, on average, between 24 and 28 weeks of pregnancy, when an oral glucose tolerance test (OGTT) is usually conducted [1]. This definition does not exclude the possibility that unrecognized glucose intolerance may have antedated or begun concomitantly with the pregnancy [1]. The incidence of GDM has increased in recent years due to the increase in reproductive age, obesity, and the implementation of more effective screening methods for GDM [2,3,4]. In Romania, the prevalence of GDM is 5.78% [5]. The most important risk factors associated with the occurrence of GDM are advanced maternal age, previous history of GDM, family history of type 2 diabetes mellitus (T2DM), ethnicity, pre-pregnancy obesity [3], excessive weight gain in the first half of pregnancy, and hypothyroidism [4,6]. From a physiopathological perspective, GDM is characterized by β-cell dysfunction (impaired insulin secretion) and insulin resistance (decreased insulin-stimulated glucose uptake in skeletal muscle and adipose tissue), like T2DM [3,7].

GDM is associated with short-term complications such as an increased risk of premature birth, cesarean delivery, macrosomia, infant born large for gestational age, low one-minute APGAR score, respiratory distress syndrome, neonatal jaundice, or admission to the neonatal intensive care unit [8], as well as with long-term maternal complications such as the occurrence of metabolic syndrome [9], T2DM [10,11], myocardial infarction, coronary artery disease [11], and for the newborn, an increased risk of childhood obesity [12], metabolic syndrome [9], and obesity and T2DM in childhood and adulthood [13,14].

The role of chronic low-grade inflammation and activation of the immune system in the occurrence of insulin resistance, T2DM, and GDM in obese patients is recognized [15,16]. Various markers of inflammation, such as white blood cell count, C-reactive protein (CRP), and various cytokines (tumor necrosis factor (TNF), interleukin (IL)-1β, and IL-6), can be used to evaluate the inflammatory status [15,16]. Other readily available markers of inflammation, such as the neutrophil-lymphocyte ratio (NLR), are associated with GDM [17].

Among the anthropometric measurements, BMI, MUAC, and TST could be used to diagnose obesity in mother and newborn couples [18].

Adipose tissue, found in excess in obese patients, is the source of numerous hormonally active substances, including cytokines (tumor necrosis factor α-TNFα, IL6, and IL34) and adipokines (adiponectin, chemerin, leptin, resistin, visfatin, etc.) [13]. The alteration of serum adipokine values found in obese patients is involved in decreased insulin sensitivity of tissues, inflammation, and the development of atherosclerosis, T2DM, psoriasis, and diabetic foot [19].

Adiponectin is one of the first adipokines discovered. It is a 30-kDa protein, structurally like complement factor C1q, secreted into the blood by white adipose tissue [20]. The adiponectin gene was located on chromosome 3q27 [21]. It is secreted in the blood in three multimeric forms: a trimeric (low molecular weight form), a hexameric (medium molecular weight form), and a high-molecular-weight form [22]. It exerts its effects on the cellular level through several receptors, including AdipoR1, AdipoR2, and T-cadherin. These receptors are mainly found in organs such as the liver, muscle, adipose tissue, kidney, heart, bone, and brain [23]. Adiponectin mostly has an insulin-sensitizing, anti-inflammatory, angiogenic, and vasodilatory effect at the brain level; at the liver level, it plays a role in controlling glucose and lipid metabolism, reduces gluconeogenesis, and stimulates glycolysis and fatty acid oxidation; at the muscle level, it stimulates insulin sensitivity and favors glucose uptake and fatty acid oxidation [23]. The level of adiponectin is low in conditions associated with obesity and an increased level of inflammation, such as in patients affected by GDM [24,25], T2DM [26], colorectal cancer [27], ovarian cancer, and endometrial [28] or cardiovascular diseases [29]; or paradoxically, it is increased in some patients with cardiovascular and renal diseases [29,30]. Thus, adiponectin can be used as a biomarker with which to diagnose these conditions. Additionally, levels of maternal adiponectin are inversely associated with birth weight [31].

Adipolin/C1qdc2/CTRP12 is a new adipokine from the C1q/TNF-related protein family (CTRP) discovered by Enomoto et al. in 2011 [32] and is encoded by the FAM132A gene located on chromosome 1p36 [32,33]. It is mainly secreted by white adipose tissue and has anti-inflammatory and insulin-sensitizing effects [32]. The serum level of adipolin is decreased in rodent obesity models [32]. In humans, the circulating level of adipolin is low in patients with T2DM [34], with polycystic ovarian syndrome (PCOS) [35], and with coronary artery disease [36]. Although adiponectin has been intensively studied in patients with gestational diabetes, adipolin has not been studied at all in these patients.

This study aimed first to investigate the serum level of adipolin and adiponectin in pregnant women with gestational diabetes and healthy women at the time of the OGTT, prepartum, and in the newborns of these patients; second, it aimed to evaluate the relationship of these adipokines with anthropometric markers (BMI, MUAC, TST), inflammation markers (NLR, PLR, CRP), and maternal glucose homeostasis parameters (fasting glucose, insulin, C peptide, HbA1c, IR HOMA); and third, it aimed to find associations between these adipokines and adverse events during pregnancy (pre-term labor, gestational hypertension, pre-eclampsia, macrosomia, polyhydramnios) and the delivery mode (vaginal or cesarean section).

## 2. Materials and Methods

### 2.1. Study Design

We conducted a prospective case–control study on a consecutively selected sample of mothers and their newborns admitted to Obstetrics and Gynecology Clinic 2 in Târgu Mureș between January 2022 and November 2023.

The inclusion criteria were pregnant women aged between 18 and 46 years, gestational age between 24 and 28 weeks of gestation (WG), singleton pregnancy with OGTT performed at 24–28 WG, compliance with follow-up conditions, and delivery at the Obstetrics and Gynecology Clinic 2 Târgu Mureș. The exclusion criteria were known patients with diabetes type 1, T2DM, GDM diagnosed before 24–28 weeks of pregnancy, polycystic ovary syndrome, acute or chronic infectious diseases, blood transfusions, hematological diseases during pregnancy, pregnancies with chromosomal or fetal malformations, multiple pregnancies, intrauterine fetal death, patients who refuse to participate to the study or did not comply with the follow-up conditions, and lack of informed consent.

Before enrollment in the study, the patients signed an informed consent form for themselves and their newborns.

After applying the inclusion and exclusion criteria, we included 165 pregnant women in the study, divided into two groups based on OGTT results: 55 women with GDM and 110 healthy pregnant women. After OGTT, all pregnant women were scheduled for appointments every 2 weeks or sooner if clinically indicated until delivery as part of routine prenatal care.

### 2.2. Diagnosis of Gestational Diabetes

Gestational age was determined using the date of the last menstrual period and confirmed by a first-trimester ultrasound in all cases. Demographics (maternal age, smoking, first-degree family history of T2DM) and medical and reproductive history were obtained through structured questionnaires.

The glucose tolerance test was performed at 24–28 WG with 75 g of orally administered glucose dissolved in 200 mL of water after 8 h of fasting, according to the International Association of Diabetes in Pregnancy Study Groups (IADPSG, 2010) recommendation [37].

Blood was collected between 8 and 10 a.m. using the classic peripheral blood collection technique. The criteria of the International Association of Diabetes in Pregnancy Study Groups (IADPSG, 2010) were used to diagnose gestational diabetes [37]. One or more of the following glycemic values (≥92 mg/dL (5.1 mmol/L) fasting; ≥180 mg/dL (10 mmol/L) at 1 h; ≥153 mg/dL (8.5 mmol/L) 2 h after glucose ingestion) have been used for the diagnosis of gestational diabetes [37]. Normal glucose tolerance was diagnosed when all OGTT glycemic values were below IADPSG thresholds.

Blood glucose was measured via spectrometry using the Atellica Solution CH 930 device (Siemens Healthcare GmbH, Forchheim, Germany).

After being diagnosed with gestational diabetes, all 55 patients were offered diet therapy (1600–1800 kcal/day with 35–40% carbohydrates) and 30 min of daily moderate-intensity aerobic exercise as part of their treatment based on the American Diabetes Association recommendations from 2023 [38]. Mothers diagnosed with GDM were instructed to monitor their fasting, one-hour, and two-hour postprandial glucose levels daily during the first two weeks after diagnosis. The therapeutic target was to maintain fasting glucose levels below <95 mg/dL (<5.3 mmol/L), below 140 mg/dL (<7.8 mmol/L) one hour after eating, and below 120 mg/dL (<6.7 mmol/L) two hours after eating. However, after two weeks of following the prescribed diet and exercise regimen, 12 patients (21.81%) still had not reached their therapeutic targets. As a result, insulin therapy with basal insulin (0.7–0.9 units/kg of body weight) was initiated based on the recommendation made by a diabetologist to help manage their blood glucose levels. Subsequent daily glucose self-monitoring was made according to the diabetologist’s recommendations.

### 2.3. Anthropometric Measurements

The variables observed in pregnant women included weight, height, MUAC, TST, and BMI at 24–28 WG and birth, weight gain between the onset of pregnancy and 24–28 WG, and total weight gain during pregnancy. The patient’s prepregnancy weight, reported at the first prenatal visit, was used to calculate her pre-pregnancy BMI.

The patient’s height (cm) was measured without shoes using a wall tape measure. The obtained value was estimated to the nearest 1 mm.

A Beurer PS digital scale (Beurer Gmbh, Ulm, Germany) was used to measure the patient’s weight (kg), subtracting 0.5 kg from the weight of the patient’s clothing. The body mass index was calculated by dividing the patient’s weight by the square of the height (kg/m^2^).

In the first hour after birth, newborns were measured for weight, length, MUAC, and TST. All pregnant women and newborns included in the study were measured.

Newborns’ weight was measured using the U-Grow electronic baby scale, U001-BS (Guangzhou Berrcom Medical Device Co., Ltd., Guangzhou, China), and length using an inextensible tape measure.

MUAC was measured halfway between the acromion and olecranon of the posterior left upper arm using an inextensible tape measure. We used a Harpenden Skinfold Caliper (Baty International, West Sussex, UK) calibrated to the nearest 0.2 mm to measure TST. The measurements were performed in the same place where the MUAC measurement was also performed. Two measurements were taken, and their mean was calculated and recorded [39].

### 2.4. Laboratory Parameters

Maternal blood samples were taken upon enrollment in the study at 24–28 WG when OGTT was conducted and at the admission hospital in the prepartum period. Umbilical cord blood was collected via puncture immediately after clamping of the umbilical cord after birth. Laboratory tests were performed in all cases in 60 min after collection.

From maternal blood, we assessed at 24–28 WG the white blood cells (WBC), neutrophils (N), lymphocytes (L), platelets (P), median platelet volume (MPV), CRP, fasting glucose level, 1 h glucose level, 2 h glucose level after 75 g of orally administered glucose, glycosylated hemoglobin A1c (HbA1c), insulinemia, C peptide value, and IR HOMA. At the admission hospital in the prepartum period, we assessed maternal blood WBC, N, L, P, MPV, CRP, glucose level, HbA1c, insulinemia, C peptide value, and IR HOMA.

The automated hematology analyzer Mindray BC 6000 (Shenzhen Mindray Bio-Medical Electronics Co., Ltd., Shenzhen, China) was used to evaluate the blood count parameters. C-reactive protein (CRP) values were determined via turbidimetry with the Atellica Solution CH 930 device (Siemens Healthcare GmbH, Germany).

For the assessment of glucose homeostasis, we evaluated the values of blood glucose via spectrophotometry, glycosylated hemoglobin via turbidimetry, insulinemia and C peptide via chemiluminescence, and the IR HOMA was estimated according to the following formula: [fasting insulin (mU/L) × fasting glucose (mmol/L)]/22.5. The Atellica Solution CH 930 device (Siemens Healthcare GmbH, Germany) was used for these investigations.

We assessed adipokines levels from maternal blood at 24–28 WG and prepartum admission in the hospital. Immediately after birth, we collected umbilical cord blood via puncture to assess neonatal adipokine levels.

The blood samples were left in a serum separator tube at room temperature for 30 min to allow the serum to clot. The blood samples were centrifuged at 6000 rev/min for 4 min at room temperature. The serum was then separated and stored at −20 °C until assayed.

Adiponectin and adipolin/CTRP 12 were tested via an automated enzyme immunoassay analyzer (DYNEX DSX Automated ELISA System, DYNEX Technologies Inc., Chantilly, VA, USA) using ELISA kits: Human total Adiponectin/ACRP30, PDRP 300, R&D Systems, (Bio-techne, Minneapolis, MN, USA) for adiponectin, and Human C1QTNF12 (C1q and Tumor Necrosis Factor Related Protein 12), NBP2-70032, Novus Biologicals, (Bio-techne, Centennial, CO, USA) for adipolin, following the manufacturer’s protocol. The intra-assay coefficient of variation for adiponectin was <4.8%, and the inter-assay coefficient of variation was <7.0%; for adipolin, the intra-assay coefficient of variation was <6.0%, and the inter-assay coefficient of variation was <5.13%. According to the manufacturer, the sensitivities of the assays were 0.246 ng/mL for adiponectin and 46.88 pg/mL for adipolin.

We recorded gestational age at birth, mode of delivery, anthropometric measurements of the mother and newborn described above, adverse events during pregnancy (preterm labor, gestational hypertension, pre-eclampsia, macrosomia, polyhydramnios) and at birth (abdominal delivery, failure of labor induction, fetal distress, perineal tears).

### 2.5. Statistical Analysis

Statistical analyses were performed using GraphPad Prism version 9.0 (GraphPad Software, Boston, MA, USA). Continuous variables were expressed as mean ± standard deviation and median (IQR). Categorical variables were expressed as percentages. The Student’s *t*-test was applied for normally distributed continuous variables, and we used the Mann–Whitney test for non-normally distributed data. The chi-square test for categorical variables was used to compare clinical characteristics between subjects with GDM and control. Pearson’s and Spearman’s correlation coefficients were calculated to determine the association of adiponectin and adipolin with anthropometric markers of obesity and inflammation markers. A two-sided *p* < 0.05 was considered statistically significant.

We conducted a priori power analysis with the program G Power Version 3.1.9.6 from Faul et al. [40] using data from Siddiqui et al. [41]. Based on these data, we estimated a medium effect size of 0.45, assuming a two-tailed *t*-test with at least 80% power and alpha = 0.05. The total number of 146 patients will be the minimum required to sample for sufficient power (n = 97) in the control group and n = 49 in the GDM group. We assumed that 165 patients divided into 110 control patients and 55 patients with GDM (2:1 ratio) would be enough for our study to have sufficient power. We analyzed GDM patients with diet and insulin therapy together as we know from the work of Mazaki-Tovi et al. [42] that there is no difference in the adiponectin levels between patients who are treated with insulin and those who are managed with diet alone in reaching glucose control.

This study has been approved by the Ethics Committee of the University of Medicine, Pharmacy, Science and Technology G E Palade Târgu Mureș (decision number: 1557/2022). It was designed and conducted according to the principles of the Declaration of Helsinki (1964).

## 3. Results

### 3.1. Demographic, Anthropometric Characteristics, and Laboratory Results at 24–28 Weeks of Pregnancy

In our study, we found no significant difference in patients’ age (*p* = 0.12), gestational age at OGTT performance (*p* = 0.21), or smoking during pregnancy (*p* = 0.19). Patient characteristics are shown in Table 1.

Patients with GDM had a higher heredocolateral history of T2DM than patients in the control group (*p* = 0.0003). Regarding the anthropometric characteristics, higher pre-pregnancy BMI and BMI at diagnosis were identified in the patients with GDM as compared to the control group (*p* < 0.0001), but gestational weight gain until OGTT was lower (*p* = 0.12). MUAC and TST were higher (*p* < 0.0001) in patients with GDM than in the control group.

Regarding the studied inflammatory markers, only white blood cells and neutrophil count were higher in patients with GDM compared to the control group (*p* = 0.02), and the rest of the studied markers were the same as in the control group.

Regarding glucose homeostasis and insulin resistance parameters, they are higher in patients with GDM compared to controls (*p* < 0.0001). In the GDM group, at the time when OGTT was conducted, there were no patients on insulin therapy.

Adipolin and adiponectin levels were lower in patients with GDM than in controls (*p* = 0.65 and *p* = 0.0009, respectively) (Table 1).

### 3.2. Demographic, Anthropometric Characteristics, and Laboratory Results at Prepartum

Gestational age at prepartum was lower in patients with GDM than in controls (*p* = 0.006). The BMI of patients with GDM was found to be higher (*p* < 0.0001) than that of patients in the control group at prepartum. Furthermore, patients with GDM had a lower total weight gain (*p =* 0.001) during pregnancy compared to the control group. MUAC and TST measurements at prepartum were higher in patients with GDM compared to control patients (*p* < 0.0001 and *p* = 0.001, respectively). Patient characteristics are shown in Table 2.

There was no significant difference between the values of the inflammatory markers neutrophil-to-lymphocyte ratio (NLR) (*p* = 0.18), platelet-to-lymphocyte ratio (PLR) (*p* = 0.45), and C reactive protein (CRP) (*p* = 0.68) of the two groups at prepartum.

In patients with GDM, glucose homeostasis and insulin resistance parameters were higher than in patients from the control group (fasting glucose level (*p* < 0.0001), insulin level (*p* = 0.005), C peptide level (*p* = 0.01), HbA1c (*p* < 0.0001), IR HOMA (*p* = 0.0004)).

The serum levels of adipolin and adiponectin at birth in patients with GDM were lower than in patients in the control group (*p* = 0.50 and *p* = 0.02, respectively).

### 3.3. Evolution from OGTT to Prepartum of Studied Parameters

From OGTT time to prepartum, regarding anthropometric parameters, both groups had a significant increase in weight, BMI (*p* < 0.0001), and TST (*p* < 0.0001); MUAC had a significant increase (*p* < 0.0001) only in control patients.

Regarding the inflammatory markers studied, the level of white blood cells (WBC) increased in patients with GDM (*p* = 0.07) and control patients (*p* < 0.0001), neutrophils (N) count increased in patients with GDM and control patients (*p* = 0.02 and *p* < 0.0001, respectively), lymphocytes (L) count increased in both groups (*p* = 0.01 and *p* = 0.008, respectively), platelets (P) count increased in patients with GDM (*p* = 0.61) and decreased in control patients (*p* = 0.02), and median platelet volume (MPV) rose substantially in both groups of patients (*p* < 0.0001). The NLR value increased in patients with GDM (*p* = 0.91) and the control group (*p* = 0.01). The PLR value decreased in both patient groups (*p* = 0.008 for the GDM group and *p* = 0.001 for the control group). The CRP value did not increase significantly in patients with GDM (*p* = 0.13) or the control group (*p* = 0.05).

Regarding glucose homeostasis, glycemia at birth in patients with GDM was lower than at the time of OGTT (*p* = 0.02), and glycemia at birth in patients in the control group was higher than at the time of OGTT (*p* = 0.03). The serum insulin level in patients with GDM was higher at birth. Still, it was not significant (*p* = 0.24); in patients in the control group, it was significantly lower (*p* = 0.02). The C peptide level decreased in patients with GDM (*p* = 0.08) but increased in patients in the control group (*p* < 0.0001). The HbA1c level increased in both groups of patients (*p* < 0.0001).

The level of IR HOMA increased in patients with GDM (*p* = 0.49) and the control group (*p* = 0.01).

The level of adipolin in patients with GDM increased (*p* = 0.67). In the control group, the level of adipolin decreased (*p* = 0.50). On the other hand, the adiponectin level at birth was significantly higher than the level during the OGTT in both groups (*p* = 0.0006 for the GDM group and *p* = 0.0049 for the control group).

### 3.4. Correlation between Clinical and Paraclinical Parameters of GDM Patients

In correlation analysis, the adipolin level in the control group correlates negatively with the BMI value at 24–28 weeks, the TST value, and the CRP level at birth; otherwise, we found no correlations between the adipolin value in the control patients and the other studied parameters. Table 3 shows the correlation between adipolin and adiponectin values and the parameters studied in both groups.

Within the control group, the adiponectin level correlates negatively with BMI and MUAC at 24–28 weeks and birth, negatively with TST at 24–28 weeks, and positively at birth and positively with the level of NLR and PLR at 24–28 weeks. The adiponectin level correlates negatively with insulin level, C peptide, IR HOMA at 24–28 weeks, and birth and negatively with HgA1c level.

In the GDM group, the adipolin value correlates positively with the HgbA1c value at birth; otherwise, we found no correlation between the adipolin value and the parameters studied.

The adiponectin value correlates negatively with BMI, MUAC, and TST values at 24–28 weeks and birth. Also, the maternal adiponectin value correlates negatively with the insulin value, C peptide, and IR HOMA at 24–28 weeks.

The patients who required insulin therapy had a higher value of adiponectin at 24–28 weeks, but it was statistically insignificant (5836 ± 3339 vs. 5125 ± 1708 ng/mL; *p* = 0.50). On the other hand, the value of adipolin was also statistically negligible but lower in patients with insulin therapy (4827 ± 1695 vs. 5468 ± 751.5 pg/mL; *p* = 0.33). There were no statistical differences between the values of adiponectin and adipolin at birth in patients with GDM who were treated with insulin vs. those who only had diet therapy (6287 ± 2230 vs. 6305 ± 3400 ng/mL, *p* = 0.98; 5577 ± 800.5 vs. 5316 ± 1032 pg/mL, *p* = 0.58, respectively) (Table 3).

### 3.5. Adverse Pregnancy Results and Delivery Mode

During the pregnancy, the following adverse results were recorded: preterm labor, gestational hypertension, pre-eclampsia, macrosomia, and polyhydramnios. In the GDM group, twenty-two (40%) adverse effects were registered: four preterm births, ten cases with gestational hypertension, one case with polyhydramnios, and ten neonates with macrosomia. In the control group, there were seventeen (15.45%) adverse events: seven preterm births, three cases with gestational hypertension, three cases with pre-eclampsia, and five neonates with macrosomia. The difference was statistically significant between groups (*p* = 0.0008). The relative risk of occurrence of adverse events in patients with GDM compared to healthy ones was 2.15 (95% CI 1.416 to 3.182), and the odds ratio for macrosomia was 4.66 (95% CI 1.591 to 12.69). The pregnant women with GDM who gave birth to macrosomic newborns had higher values of glucose levels (104 ± 42 vs. 82 ± 11 mg/dL; *p* = 0.006), HgbA1c (6.3 ± 0.55 vs. 5.2 ± 0.38; *p* < 0.0001), insulinemia (42.38 ± 30.48 vs. 13.86 ± 14 mUI/L; *p* < 0.0001), and IR HOMA (13.3 ± 16.57 vs. 2.93 ± 3.32; *p* < 0.0001) than control patients.

There were no significant differences between the levels of adipolin and adiponectin at 24–28 weeks and birth between the groups with and without complications during the pregnancy taken as a whole, but also when they were evaluated separately by groups, i.e., the one with GDM and the control, respectively.

Regarding the delivery mode, it was observed in the control group that the levels of adipokines were higher in patients who underwent a cesarean section than in those who gave birth vaginally (*p* = 0.10 for adipolin and *p* > 0.99 for adiponectin) (Table 4). In patients with GDM, the adipolin level was higher (*p* = 0.01) in patients who gave birth via cesarean section. The level of adiponectin was not different between the groups (*p* = 0.25). In the whole group, the levels of adipolin and adiponectin were higher in patients who gave birth via cesarean section (*p* = 0.006 and *p* = 0.48, respectively). Table 4 shows the serum levels of adipolin and adiponectin at birth with respect to the route of birth.

There was no statistical difference between the cesarean section rate in the GDM group compared to the control group (35/55 vs. 72/110; *p* = 0.86). Indications for cesarean section in the GDM group included prior cesarean scar (20 cases; 57.14%), cephalopelvic disproportion (3 cases; 8.57%), failure of labor induction (3 cases; 8.57%), breech presentation (2 cases; 5.71%), fetal distress (1 case; 2.85%), and other indications (6 cases; 17.14%). In the control group, the indications for cesarean section included prior cesarean scar (33 cases; 45.83%), cephalopelvic disproportion (9 cases; 12.5%), failure of labor induction (2 cases; 2.77%), breech presentation (1 case; 1.38%), fetal distress (5 cases; 6.94%), and other indications (22 cases, 30.55%).

Regarding the incidence of perineal tears at a vaginal birth, there was no statistical difference between the GDM group and the control group (5/19 vs. 5/38; *p* = 0.27).

### 3.6. Anthropometric Characteristics and Adipokine Levels among Included Newborns

We found no difference between the groups regarding birth weight, newborn length, MUAC, and TST values. Adipolin levels in newborns from mothers with GDM were not different (*p* = 0.24) from those in newborns from control mothers. Serum adiponectin values in newborns were much higher than those in pregnant women. In newborns from mothers with GDM, the adiponectin value was lower than in newborns from control mothers (*p* < 0.0001). Table 5 shows the anthropometric characteristics and adipokine values in newborns.

In the control group, we failed to demonstrate any correlation between prepregnancy BMI, MUAC, and TST at 24–28 WG and birth and newborn weight, newborn MUAC, and TST, and between the level of adipokines at the time of diagnosis and birth and neonatal weight, length, MUAC, and TST.

In the group with GDM, we found positive correlations between the value of MUAC and maternal TST at 24–28 weeks and the weight of the newborn (r = 0.317, *p* = 0.018; r = 0.384, *p* = 0.004, respectively), newborn TST (r = 0.274, *p* =0.043; r = 0.304, *p* = 0.024, respectively), and maternal TST at 24–28 weeks and newborn MUAC (r = 0.332, *p* = 0.013).

Also, in the GDM group, we found positive correlations between maternal BMI, MUAC, and TST at birth and newborn weight (r = 0.365, *p* = 0.006; r = 0.407, *p* = 0.002; r = 0.438, *p* = 0.001); maternal MUAC and TST and newborn MUAC (r = 0.289, *p* = 0.03; r = 0.315, *p* = 0.019); and maternal BMI, MUAC, TST, and newborn TST (r = 0.311, *p* = 0.002; r = 0.424, *p* = 0.001; r = 0.410, *p* = 0.002). The correlation between maternal BMI, MUAC, and TST at birth and newborn anthropometric parameters are represented in Table 6.

We found no correlations between glucose homeostasis parameters in patients with GDM and controls and neonatal weight, length, MUAC, and TST.

Regarding the value of adipokines in the GDM group, we found a positive correlation between maternal adipolin level at 24–28 weeks and birth and neonatal adipolin (r = 0.556, *p* = 0.001; r = 0.332, *p* = 0.013, respectively). We did not find correlations between maternal adipolin and adiponectin values at the time of diagnosis and birth and newborn weight, length, MUAC, TST, and adiponectin level.

## 4. Discussion

Adipolin, an adipokine with anti-inflammatory and insulin-sensitizing effects [32], is found at low levels in patients with T2DM [34] and those with PCOS [35]. Adiponectin is one of the best-studied adipokines, whose level is low in patients with GDM [24,25] and T2DM [26], being proposed as a biomarker for these conditions. To our knowledge, this is the first time that the serum level of adipolin has been measured in healthy pregnant patients and those with GDM to evaluate its role in the occurrence of GDM.

### 4.1. Association of Demographic Characteristics with GDM

In our study, we did not find significant differences regarding the age of the patients at study enrollment. Doruk et al. [43] found that pregnant women with GDM were older than healthy pregnant women (*p* < 0.0001), but they included in their study a smaller number of patients compared to us. One explanation of our results is that our cohort contained older primiparous women in our healthy control group compared with the GDM group; and in the GDM group, we have more older multiparous pregnant women than in the control group. In their studies, McIntyre et al. [3] and Pinheiro et al. [44] found that older maternal age is a risk factor for GDM.

Regarding the family history of T2DM, we found a significant association between the presence of T2DM in first-degree relatives and the occurrence of GDM, in agreement with what McIntyre also found [3], suggesting the existence of a genetic predisposition towards this condition. On the contrary, Siddiqui et al. [41] did not find an association between the family history of T2DM and the occurrence of GDM.

We found that the pre-pregnancy BMI of patients with GDM was greater than that of the control group. Our findings suggest that greater BMI and adiposity at preconception might be risk factors for GDM occurrence rather than the mother’s age.

According to a study conducted by Jiang et al. [45], patients with GDM gave birth at a lower gestational age than control patients. Similarly, in our study, patients with GDM gave birth mainly at term but at a statistically significantly lower gestational age compared to control patients. This was primarily due to elective births at or near term to minimize perinatal complications.

### 4.2. Correlation between Anthropometric Factors and Adipokines

Doruk et al. [43] found that women with gestational diabetes were overweight before pregnancy and had significantly higher pre-pregnancy and OGTT BMI compared to control patients. Siddiqui et al. [41] found no differences concerning BMI between groups until OGTT time. According to Zhang et al. [46], having a high BMI during early pregnancy can increase the risk of developing GDM. In our study, the patients with GDM were overweight before becoming pregnant, and the control patients had a healthy weight, with the difference between the groups being statistically significant. The occurrence of GDM in our study group is mainly due to overweight and obesity and the subsequently increased insulin resistance and β-cell dysfunction induced by these conditions. Also, McIntyre et al. [3], in their review, stated that overweight and obesity before pregnancy are the most significant GDM risk factors.

Total weight gain during pregnancy in patients with GDM was significantly lower than that of control patients due to the diet treatment that they received from diagnosis of GDM to birth. Still, it was more important than weight gain, as recommended by the Institute of Medicine (5–9.1 kg for obese pregnant women) [47]. At birth, patients with GDM had a significantly higher BMI than control patients.

However, gaining weight before being diagnosed with GDM is not associated with an increased risk of the condition. This highlights the importance of maintaining a healthy weight and avoiding overweight or obesity before becoming pregnant to prevent the occurrence of GDM.

Mwanri et al. [48] found that a value of MUAC above 28 cm at OGTT time is associated with a higher risk of the development of GDM (OR 1.9; 95% CI 1.1–3.3). The patients in our group with GDM had a MUAC value at OGTT time of 30.76 ± 4.48 cm. Huidobro et al. [49] found that the TST value was statistically significantly higher in patients with GDM than in control patients when performing the OGTT. In our study, MUAC and TST, as a mirror of subcutaneous adipose tissue, were significantly higher in patients with GDM than in controls at the time of OGTT and birth. During pregnancy, there was a significant increase in these parameters from the time of OGTT to birth. Cremona et al. [50] developed a risk prediction model that incorporated a family history of diabetes, previous perinatal death, the overall insulin-resistant condition, weight, abdominal subcutaneous tissue (SAT), abdominal visceral tissue (VAT), eight-point skinfold thickness (SFT), and MUAC. The prediction model achieved a good level of discrimination in the first trimester of pregnancy, with an AUC of 0.860 (CI 0.774–0.945) for GDM. Also, Salmen et al. [51] showed that ultrasound assessment of VAT in the first trimester, alone or in conjunction with other clinical and biological parameters, may predict the occurrence of GDM later in pregnancy. Thus, based on these results and our findings, MUAC and TST can be useful and easy-to-obtain parameters that, combined with other parameters like ultrasound assessment of VAT in the first or second trimesters of pregnancy, can predict the occurrence of GDM.

Regarding the correlation between adipokines and anthropometric parameters, Shanaki et al. [35] found that in non-PCOS patients, adipolin was inversely correlated with BMI. Still, it did not correlate with BMI in PCOS patients. Doruk et al. [43] found negative correlations between adiponectin values, pre-pregnancy BMI, BMI at GDM diagnosis, and the whole group. Thagaard et al. [52] found lower values of adiponectin in the first trimester of pregnancy in patients with GDM in all BMI groups. Kapustin et al. [53] found that adiponectin negatively correlated with BMI in the first and third trimesters of pregnancy. We found that the serum adipolin value correlates negatively with BMI at 24–28 weeks and with TST at birth in control patients. We found no correlations between serum adipolin values and BMI, MUAC, and TST in patients with GDM. The adiponectin value correlates significantly negatively with BMI, MUAC, and TST in control and patients with GDM at 24–28 weeks and birth (except TST at birth in GDM patients). In accordance with the findings of the above-mentioned researchers, our findings suggest that lower adiponectin values during the second trimester and prepartum period in patients GDM are due to the greater BMI, greater adiposity, and, in consequence, the greater insulin resistance of these patients and the subsequent alteration of adiponectin homeostasis. Still, we cannot find these associations in the case of adipolin in GDM patients.

### 4.3. Correlation between New Inflammatory Markers and Adipokines

Wang et al. [54] and Liu et al. [55] found that NLR and PLR were higher in patients with GDM at diagnosis, but Hessami [17] found in their meta-analysis that only NLR was associated with GDM, suggesting a role for inflammation in the onset of gestational diabetes. Sargin et al. [56] found no statistically significant differences between the groups regarding NLR and PLR at the time of diagnosis. Our study found no statistically significant differences between the groups concerning the studied inflammatory markers, neither at the time of diagnosis nor at prepartum. In conclusion, they cannot be used as a predictor of GDM. An explanation could be that the two groups presented no significant differences in terms of WBC, N, and PLT values.

Savard et al. [57] did not observe a variation in CRP values in normal pregnancy across trimesters. Jiang et al. [58] found higher CRP values in patients with GDM. Alamolhoda et al. [59] found that an increased CRP value in the first trimester of pregnancy can be a risk factor for GDM. In our study, the mean values of CRP in both groups were higher than the average values for the second and third trimesters [60]. The increase in serum CRP values throughout pregnancy in control patients was statistically significant; still, in patients with GDM, it was not. This increase in CRP values in control patients during pregnancy could be related to the significantly higher gestational weight gain in this group than in patients with GDM (15.55 ± 5.40 vs. 12.48 ± 6.48; *p* = 0.001).

In the control group, we found positive correlations between the values of NLR, PLR, and adiponectin at the time of diagnosis, and a positive correlation between the value of CRP at birth and adipolin. In the GDM group, we did not find correlations between the values of the studied inflammatory markers and the adipokines, neither at the time of diagnosis nor at the time of birth.

### 4.4. Correlation of Glucose Homeostasis Markers and Adipokines

Kapustin et al. [53] found that adiponectin was negatively correlated with OGTT levels. Vitoratos et al. [61] found that adiponectin was negatively correlated with IR HOMA during pregnancy in GDM patients. At diagnosis, Altinova et al. [62] found negative correlations between adiponectin value, fasting glucose level, and IR HOMA. We found statistically significant negative correlations between the value of adiponectin in control patients at diagnosis and at birth and the value of insulin, C peptide, and IR HOMA, and between the value of adiponectin and HbA1c at the time of diagnosis. These findings are related to insulin resistance induced by gestation weight gain during healthy pregnancy. We did not find significant correlations between markers of glucose homeostasis and adipolin value in the control patients.

In patients with GDM, we found statistically significant negative correlations between the adiponectin value and the insulin value, C peptide, and IR HOMA at the time of diagnosis and birth. However, we found a statistically significant positive correlation with the HbA1c level at birth regarding adipolin. These findings are related to the obesity-induced alteration of glucose and adipose tissue homeostasis, which results in a subsequent lowering of the adiponectin level.

### 4.5. Adipokines and GDM

Bai et al. [29] discovered that individuals diagnosed with T2DM exhibited significantly lower adipolin levels. This suggests that adipolin could be a new biomarker for early diagnosis and prediction of T2DM. Shanaki et al. [35] showed that adiponectin and adipolin levels were significantly lower in normal-weight and overweight/obese PCOS women compared to the corresponding non-PCOS women, possibly due to adipose tissue dysfunction secondary to aberrant adipocyte morphology and different gene expression profiles.

Our study showed no statistical differences between the groups regarding the adipolin level at diagnosis and birth. The adipolin value did not change significantly during pregnancy. Because of these findings, we cannot conclude that adipolin has a role in the occurrence of GDM. Further studies on larger groups of patients are probably needed in order to observe significant changes in this adipokine in GDM patients.

Yuan et al. [63] found statistically significantly lower adiponectin values in patients with GDM compared to control patients at 16–18 weeks gestational age and an AUROC value of 0.751. Thagaard et al. [52] concluded that low adiponectinemia in the first trimester is associated with the development of GDM. On the other hand, Florian et al. [64] did not find a significant association between the adiponectin value in the first trimester of pregnancy and the subsequent development of GDM. These two research groups reached different conclusions because Thagaard et al. [52] had a large number of obese patients with GDM, and Florian et al. [64] had normal-BMI patients with GDM in the first trimester. Ianniello et al. [65] found that overweight and obese patients with GDM have a lower adiponectin value than control patients with the same BMI during all three trimesters. These data and those we found suggest the role of low adiponectin in GDM occurrence. In our study, adiponectin was statistically significantly lower in patients with GDM than control patients at diagnosis and birth.

Fuglsang et al. [66] found that adiponectin levels decrease during normal pregnancy and are inversely associated with BMI. Kapustin et al. [53] found that adiponectin levels in patients with GDM decrease with advancing pregnancy and negatively correlate with BMI. Randeva et al. [67] noted higher adiponectin levels in T1DM throughout gestation compared to normal pregnancies without explaining this finding. Zhao et al. [29] showed that in patients with severe cardiovascular disease or chronic liver disease, the increased levels of adiponectin are more due to delayed clearance in the context of increased adipose tissue inflammation. Our study found that adiponectin levels increased significantly during pregnancy in both groups. Such a mechanism of delayed clearance could be responsible for our study’s increase in adiponectin values at prepartum.

Fadaei et al. [36] found a significantly lower serum level of adipolin in patients with coronary artery disease than control patients, suggesting a possible link between adipolin and the pathogenic mechanisms of atherosclerosis, such as inflammation and high-density lipoprotein–cholesterol metabolism. In the GDM group, we found no association between adipolin levels and the studied inflammatory markers.

Doruk et al. [43] found that patients with GDM had a 4.6 relative risk of adverse events during pregnancy and that adiponectin levels at 24–28 weeks were statistically significantly lower in patients with adverse events taken as a whole. Vieira et al. [68] determined that higher adiponectin levels in combination with multiparity, lower maternal age, systolic blood pressure, and HbA1c levels at 15 + 0 to 18 + 6 weeks’ gestation were associated with uncomplicated pregnancy and birth in obese women. Abraham et al. [69] found that the level of adiponectin was significantly increased in patients with pre-eclampsia and impaired renal function compared to the control group and compared to the group with gestational hypertension in pregnant women between 28 and 42 weeks of pregnancy. Masuyama et al. [70] found that adiponectin was increased only in patients with late-onset pre-eclampsia. Lekva et al. [71] found an association between the birth of LGA infants and low adiponectin levels at the beginning of the second trimester, which continued to decrease throughout the pregnancy. Savvidou et al. [72] did not find significant differences between the level of adiponectin and leptin in the second trimester of patients who subsequently develop pre-eclampsia and/or fetal growth restriction compared to those who underwent a normal pregnancy. In our study, in patients with GDM, we observed a relative risk of adverse events of 2.15 (95% CI 1.416 to 3.182). Our study showed no statistical differences between the levels of adipolin and adiponectin in patients with and without adverse events during pregnancy at diagnosis and birth. The differences between the conclusions of the various studies related to the association between the level of adiponectin and adverse pregnancy outcomes may be generated by the differences associated with gestational age at blood collection, the BMI of the patients, and the number of patients included in the study.

Doruk et al. [43] found a relative risk of cesarean delivery of 1.6 in patients with GDM and a statistically significant lower level of adiponectin in the entire cesarean group. Fazeli et al. [73] found that the maternal adiponectin serum level was significantly higher in the vaginal delivery group compared to the cesarean section group in a healthy pregnant women group. Our study showed no statistical differences in the cesarean rate between the two groups nor the incidence of perineal tears. The level of adipolin at delivery was significantly higher in patients with GDM who delivered via cesarean section and in the whole cesarean delivery group. The level of adiponectin at birth did not differ between groups concerning the mode of delivery.

### 4.6. Newborn Characteristics and Adipokines

Teshome et al. [74] found no differences in weight and length between newborns born to mothers with GDM and those born to healthy mothers. We found no statistical differences between the weight, length, MUAC, and TST of the newborns born from mothers with GDM and those from healthy mothers. The reason could be that all pregnant women with GDM received treatment during pregnancy, such as diet or insulin therapy, and their glucose values were optimally controlled.

Pirc et al. [75] found that the cord adiponectin level was lower in the GDM than in the control group. We also found that the adiponectin level of newborns born from mothers with GDM was significantly lower than that of newborns born from healthy mothers, and the neonatal adiponectin level was significantly higher than the maternal level. The explanation for a lower level of newborn adiponectin from mothers with GDM compared with control ones could be based on the alteration of adipose tissue homeostasis in the presence of greater adiposity.

In their study, Mărginean et al. [18] found that birth weight, newborn MUAC, and TST are significantly associated with gestational weight gain status. Santos et al. [76] found that excessive weight gain during pregnancy was associated with an increased risk for macrosomia in women with GDM. Yang et al. [77] found an odds ratio for macrosomia of 2.13 (95% CI 1.34–3.40). We found an odds ratio for macrosomia of 4.66 (95% CI 1.591 to 12.69) in the GDM group. This was due to higher levels of glucose and HbA1c in pregnant women with GDM compared to control patients, despite antidiabetic treatment (*p* = 0.006 and *p* < 0.0001, respectively).

Chen et al. [78] discovered positive correlations between infant birth weight and pre-pregnancy BMI, prepartum BMI, and gestational weight gain. In our study, in the GDM group, we found positive correlations between maternal MUAC at diagnosis and newborn weight and TST and between maternal TST and newborn weight, MUAC, and TST. At birth, we found significant positive correlations between maternal BMI and newborn weight and TST, maternal MUAC and TST, and newborn weight, MUAC, and TST. Lekva et al. [71] found that maternal adiponectin may significantly predict fetal growth and birth weight, independent of BMI and insulin resistance, in healthy mothers and mothers with GDM. On the contrary, Fazeli et al. [73] found no significant correlation between fetal sex and anthropometric measurements with maternal adiponectin and umbilical cord blood adiponectin. Also, Bomba-Opon et al. [79] found no association between adiponectin, leptin, fasting insulin, and IR HOMA in the third trimester of pregnancy and neonatal birth weight or birth weight percentile. Chen et al. [78] found that neonatal birth weight and a one-year-old infant’s weight correlate with the cord blood adiponectin level. In patients with GDM, we found correlations between the serum adipolin value at the time of diagnosis and birth and the neonatal adipolin value. We found no correlations between maternal adipolin and adiponectin levels at diagnosis and birth in both groups and newborn weight, length, MUAC, TST, and adiponectin level. The differences in the results of these studies and our results may be due to differences in fetal weight by race or the varying management of GDM between clinics.

What was known: Adiponectin is lower in pregnant women with GDM compared to healthy women at the time of diagnosis and later during pregnancy; newborns of mothers with GDM have lower adiponectin values than newborns of healthy mothers.

What was not known: The adipolin level in the second trimester and prepartum period in women with GDM and healthy patients, as well as the adipolin level in neonates of women with GDM and healthy mothers.

What is new: The level of adipolin in pregnant women with GDM during the second trimester and prepartum period does not differ from that of healthy pregnant women. Therefore, it cannot be concluded that adipolin is involved in the pathogenesis of GDM or that it can be used as a biomarker of GDM. The neonatal adipolin level of newborns of mothers with GDM did not differ from that of newborns of healthy mothers. The adipolin level of pregnant women with GDM at diagnosis and prepartum correlates with the neonatal adipolin level. The level of adiponectin increased from the time of diagnosis to the time of prepartum in both patients with GDM and healthy patients.

The practical implication of our study is that we cannot use adipolin in the second trimester as a predictor of GDM, but we can use adiponectin as a biomarker of this condition. Further research directions could include evaluating the level of adipolin in patients with GDM later in life as a predictor of T2DM occurrence as there is evidence that the adipolin level is lower in patients with T2DM [29], and it is known that one of the long-term complications of GDM is T2DM [10,11].

The findings of this study can be extended to other populations of healthy pregnant women and patients with GDM with similar anthropometric and medical characteristics.

We want to acknowledge that our study has some limitations, as follows:

1. The sample size of patients in our study is relatively small, which may have reduced the statistical power of our research.
2. We did not analyze the patients’ diets or assess the level of adipolin and adiponectin in the first trimester.
3. We did not match the patients for BMI.

Our study also has several strengths:

1. We collected data on pregnancy and birth with great accuracy. We calculated the BMI using measured weight and height during the second trimester and at birth rather than self-reported weight and height.
2. We assessed mothers’ and newborns’ clinical, laboratory, and anthropometric parameters. We also measured adipokine levels during pregnancy, in the second trimester, at birth, and in newborns.
3. This is the first study conducted in Romania to assess the level of adipolin in healthy pregnant women and women with GDM and their newborns.

## 5. Conclusions

Our study revealed that the serum values of adipolin in the second trimester and prepartum in pregnant women with GDM are not different from those of healthy ones. It seems that adipolin does not play a role in the occurrence of GDM. Therefore, it cannot be used as a biomarker of GDM. The level of neonatal adipolin was also not different between the groups or compared to the maternal prepartum level. However, adipolin was found to be higher in patients with GDM who delivered via cesarean section compared to those who delivered vaginally.

We also found that pregnant women with GDM had a lower level of adiponectin at the time of diagnosis and prepartum, indicating the importance of adiponectin in developing GDM. The level of adiponectin increased during pregnancy in both groups of patients, possibly due to delayed clearance in the context of adipose tissue inflammation secondary to high GWG during pregnancy. Additionally, newborns had higher adiponectin values than maternal adiponectin levels, and those born to mothers with GDM had lower values than those born to healthy mothers.

Further research with larger groups of patients is needed to evaluate the role of adipolin in the development of GDM fully.

## Figures and Tables

**Table 1:** Demographic, anthropometric characteristics, and laboratory results at 24–28 weeks of pregnancy.

Parameters	Control Group—24–28 Weeks (n = 110)	GDM Group—24–28 Weeks (n = 55)	*p*-Value
Age, years, Mean (SD)	30.88 ± 5.02	32.07 ± 5.12	0.12
Smoking, %	16 (14.55%)	13 (23.64%)	0.19
Heredocolateral history of T2DM, %	10 (9.09%)	18 (32.73%)	0.0003
Gestational age, weeks, Mean (SD)	25.73 ± 1.31	26.02 ± 1.45	0.21
Pre-pregnancy BMI, kg/m^2^, Mean (SD)	22.77 ± 3.77	28.36 ± 6.01	<0.0001
BMI, kg/m^2^, Mean (SD)	25.94 ± 3.65	31.08 ± 5.72	<0.0001
GWG until OGTT, kg, Mean (SD)	8.33 ± 4.12	7.21 ± 4.30	0.12
MUAC, cm, Mean (SD)	26.84 ± 3.21	30.76 ± 4.48	<0.0001
TST, mm, Mean (SD)	17.36 ± 5.89	21.76 ± 5.35	<0.0001
White blood cells, Mean (SD)	9483 ± 2121	10,243 ± 2134	0.02
Neutrophil count, Mean (SD)	6866 ± 1769	7542 ± 1920	0.02
Lymphocyte count, Mean (SD)	1889 ± 473	1991 ± 572	0.25
Platelet count, Mean (SD)	245,195 ± 65,779	249,727 ± 52,213	0.57
MPV, fl, Mean (SD)	9.84 ± 1.07	9.89 ± 1.21	0.68
NLR, Mean (SD)	3.81 ± 1.23	4,06 ± 1.51	0.49
PLR, Mean (SD)	137.3 ± 43.22	133.6 ± 40.82	0.59
CRP, mg/dL, Mean (min–max)	0.86 (0.05–21.1)	0.78 (0.05–3.3)	0.58
Fasting glucose level, mg/dL, Mean (SD)	79.75 ± 5.49	97.18 ± 13.37	<0.0001
1 h glucose level, mg/dL, Mean (SD)	124.8 ± 24.46	179.3 ± 38.68	<0.0001
2 h glucose level, mg/dL, Mean (SD)	101.8 ± 18.62	139.1 ± 39.53	<0.0001
Insulin, mUI/L, Mean (min–max)	14.59 (3.10–214.5)	24.75 (3.70–396.7)	<0.0001
C Peptide, ng/mL, Mean (SD)	2.52 ± 1.84	4.16 ± 5.05	<0.0001
HbA1c, %, Mean (SD)	4.91 ± 0.32	5.39 ± 0.56	<0.0001
IR HOMA, Mean (min–max)	2.88 (0.30–41.84)	6.33 (0.70–106.8)	<0.0001
Adipolin, pg/mL, Mean (SD)	5393 ± 587	5340 ± 1024	0.65
Adiponectin, ng/mL, Mean (SD)	6484 ± 2217	5267 ± 2114	0.0009

Note: T2DM = type 2 diabetes mellitus; BMI = body mass index; GWG = gestational weight gain; OGTT = oral glucose tolerance test; MUAC = mid-upper arm circumference; TST = tricipital skinfold thickness; MPV = mean platelet volume; NLR = neutrophil-to-lymphocyte ratio; PLR = platelet-to-lymphocyte ratio; CRP = C reactive protein; HbA1c = glycosylated hemoglobin; IR HOMA = homeostasis model of assessment for insulin resistance; SD = standard deviation.

**Table 2:** Demographic, anthropometric characteristics, and laboratory results at prepartum.

Parameters	Control Group (n = 110)	GDM Group (n = 55)	*p*-Value
Gestational age, weeks, Mean (SD)	38.73 ± 1.3	38.18 ± 1.42	0.006
BMI, kg/m^2^, Mean (SD)	28.57 ± 3.72	33.00 ± 5.74	<0.0001
GWG, kg, Mean (SD)	15.55 ± 5.40	12.48 ± 6.48	0.001
MUAC, cm, Mean (SD)	27.91 ± 2.90	31.24 ± 4.10	<0.0001
TST, mm, Mean (SD)	19.42 ± 6.14	22.24 ± 5.05	0.001
White blood cells, count, Mean (SD)	10,889 ± 2310	10,862 ± 2961	0.94
Neutrophil count, Mean (SD)	8049 ± 2174	8196 ± 2607	0.70
Lymphocyte count, Mean (SD)	2016 ± 556	2120 ± 2450	0.15
Platelet, count, Mean (SD)	236,782 ± 60,573	250,145 ± 70,952	0.17
MPV, fl, Mean (SD)	11.12 ± 1.28	11.15 ± 1.79	0.99
NLR, Mean (SD)	4.33 ± 2.03	4.09 ± 2.15	0.18
PLR, Mean (SD)	123.9 ± 45.75	120.1 ± 40.96	0.45
CRP, mg/dL, Mean (min–max)	1.01 (0.09–15.04)	0.91 (0.05–5.22)	0.68
Fasting glucose level, mg/dL, Mean (SD)	82.80 ± 11.82	93.34 ± 22.39	<0.0001
Insulin, mUI/L, Mean (min–max)	13.86 (1.9–138.5)	25.83 (3.4–128.4)	0.005
C Peptide, ng/mL, Mean (SD)	3.01 ± 1.56	3.83 ± 2.54	0.01
HbA1c, %, Mean (SD)	5.35 ± 0.38	5.74 ± 0.55	<0.0001
IR HOMA, Mean (min–max)	2.93 (0.4–31.46)	6.51 (0.61–58.1)	0.0004
Adipolin, pg/mL, Mean (SD)	5367 ± 978	5364 ± 979	0.50
Adiponectin, ng/mL, Mean (SD)	7206 ± 3498	6312 ± 3150	0.02

Note: BMI = body mass index; GWG = gestational weight gain; MUAC = mid-upper arm circumference; TST = tricipital skinfold thickness; MPV = mean platelet volume; NLR = neutrophil-to-lymphocyte ratio; PLR = platelet-to-lymphocyte ratio; CRP = C reactive protein; HbA1c = glycosylated hemoglobin; IR HOMA = homeostasis model of assessment for insulin resistance; SD = standard deviation.

**Table 3:** Spearman’s correlation analysis of maternal levels of adipolin and adiponectin with other maternal parameters in the control group and GDM group.

Adipokine		Adipolin		Adiponectin		Subgroup GDM	Adipolin		Adiponectin	
Variables	Subgroup Control	Correlation Coefficient—r	*p*-Value	Correlation Coefficient—r	*p*-Value		Correlation Coefficient—r	*p*-Value	Correlation Coefficient—r	*p*-Value
BMI	Control—24–28	−0.11	0.04	−0.30	0.001	GDM—24–28 weeks	−0.04	0.76	−0.51	0.0001
	Control—Birth	−0.15	0.10	−0.25	0.006	GDM—Birth	0.05	0.67	−0.24	0.07
MUAC	Control—24–28 weeks	−0.02	0.82	−0.23	0.01	GDM—24–28 weeks	−0.11	0.40	−0.57	0.0001
	Control—Birth	−0.16	0.07	−0.24	0.009	GDM—Birth	−0.01	0.91	−0.30	0.02
TST	Control—24–28 weeks	0.04	0.65	−0.17	0.06	GDM—24–28 weeks	−0.13	0.31	−0.50	0.0001
	Control—Birth	−0.22	0.01	0.63	0.0001	GDM—Birth	0.07	0.56	−0.16	0.2
NLR	Control—24–28 weeks	0.02	0.77	0.22	0.02	GDM—24–28 weeks	−0.04	0.77	0.03	0.98
	Control—Birth	0.04	0.96	0.084	0.38	GDM—Birth	−0.18	0.18	−0.13	0.32
PLR	Control—24–28 weeks	0.01	0.99	0.30	0.01	GDM—24–28 weeks	−0.06	0.63	0.22	0.10
	Control—Birth	−0.05	0.58	−0.09	0.34	GDM—Birth	0.05	0.70	−0.001	0.99
CRP level	Control—24–28 weeks	−0.05	0.56	−0.10	0.27	GDM—24–28 weeks	−0.13	0.33	−0.20	0.12
	Control—Birth	−0.21	0.02	−0.16	0.9	GDM—Birth	0.05	0.7	0.06	0.64
Fasting glucose level	Control—24–28 weeks	0.01	0.90	−0.15	0.11	GDM—24–28 weeks	0.07	0.59	0.004	0.97
	Control—Birth	0.13	0.15	−0.13	0.12	GDM—Birth	−0.10	0.42	−0.12	0.38
Insulin level	Control—24–28 weeks	−0.03	0.75	−0.22	0.01	DM—24–28 weeks	0.08	0.52	−0.27	0.04
	Control—Birth	−0.08	0.40	−0.25	0.008	GDM—Birth	0.19	0.15	−0.23	0.08
C Peptide level	Control—24–28 weeks	0.09	0.32	−0.21	0.02	GDM—24–28 weeks	0.13	0.38	−0.34	0.01
	Control—Birth	−0.006	0.94	−0.22	0.02	GDM—Birth	0.15	0.25	−0.25	0.06
HbA1c level	Control—24–28 weeks	0.06	0.48	−0.22	0.01	GDM—24–28 weeks	−0.19	0.15	−0.12	0.34
	Control—Birth	−0.07	0.44	−0.08	0.35	GDM—Birth	0.30	0.02	−0.08	0.56
IR HOMA	Control—24–28 weeks	−0.05	0.57	−0.26	0.005	GDM—24–28 weeks	0.07	0.57	−0.28	0.03
	Control—Birth	−0.04	0.61	−0.21	0.02	GDM—Birth	0.10	0.44	−0.26	0.04

Note: GDM = gestational diabetes mellitus; BMI = body mass index; MUAC = mid-upper arm circumference; TST = tricipital skinfold thickness and volume; NLR = neutrophil-to-lymphocyte ratio; PLR = platelet-to-lymphocyte ratio; CRP = C reactive protein; HbA1c = glycosylated hemoglobin; and IR HOMA = homeostasis model of assessment for insulin resistance.

**Table 4:** Serum levels of adipolin and adiponectin at birth to the route of birth.

Patient Group	Cesarean Section	Vaginal Delivery	*p*-Value
Control group, no, %	72 (65.45%)	38 (34.54%)	
Adipolin, pg/mL, Mean (SD)	5440 ± 966.7	5226 ± 997.3	0.10
Adiponectin, ng/mL, Mean (SD)	7376 ± 4051	6885 ± 2100	>0.99
GDM group, no, %	36 (65.45%)	19 (34.54%)	
Adipolin, pg/mL, Mean (SD)	5559 ± 1028	4995 ± 777	0.01
Adiponectin, ng/mL, Mean (SD)	6654 ± 3550	5663 ± 2138	0.25
Whole group, no, %	108 (65.45%)	57 (34.54%)	
Adipolin, pg/mL, Mean (SD)	5480 ± 984.2	5149 ± 929.2	0.006
Adiponectin, ng/mL, Mean (SD)	7135 ± 3889	6478 ± 2173	0.48

Note: GDM = gestational diabetes mellitus; SD = standard deviation.

**Table 5:** Anthropometric characteristics and adipokine levels among included newborns.

Parameters	Control Group (n = 110)	GDM Group (n = 55)	*p*-Value
Birth weight, g, Mean (SD)	3342 ± 444.2	3467 ± 547.2	0.11
Newborn length, cm, Mean (SD)	53.11 ± 2.24	53.27 ± 2.58	0.79
MUAC, cm, Mean (SD)	10.85 ± 0.93	11.17 ± 1.07	0.10
TST, mm, Mean (SD)	5.66 ± 1.38	6.23 ± 1.54	0.051
Adiponectin ng/mL, Mean (SD)	34,814 ± 36,242	19,582 ± 7738	<0.0001
Adipolin pg/mL, Mean (SD)	5603 ± 868	5704 ± 725	0.24

Note: GDM = gestational diabetes mellitus; MUAC = mid-upper arm circumference; TST = tricipital skinfold thickness.

**Table 6:** Spearman’s correlation analysis of newborn weight, MUAC, and TST with maternal BMI, MUAC, and TST at birth in the GDM group.

Maternal Parameters		BMI	MUAC	TST
Newborn weight	Spearman correlation	0.365 **	0.407 **	0.438 **
	*p*-value	0.006	0.002	0.001
Newborn MUAC	Spearman correlation	0.234	0.289 *	0.315 *
	*p*-value	0.086	0.032	0.019
Newborn TST	Spearman correlation	0.311 *	0.424 **	0.410 *
	*p*-value	0.002	0.001	0.002

Note: BMI = Body mass index; MUAC = mid-upper arm circumference; TST = tricipital skinfold thickness; * *p <* 0.05, ** *p* < 0.01.

## Data Availability

The data supporting this study’s findings are available from the corresponding author (V.S.) upon reasonable request.
